# Exploring the legitimacy of industry-led farm animal welfare governance using examples of Canadian and United States dairy standards

**DOI:** 10.1017/awf.2025.17

**Published:** 2025-03-31

**Authors:** Christine Kuo, Daniel M. Weary, Steven M. Roche, Marina A.G. von Keyserlingk

**Affiliations:** 1 Animal Welfare Program, Faculty of Land and Food Systems, The University of British Columbia, 2357 Main Mall Vancouver, BC, Canada, V6T 1Z6; 2 ACER Consulting, 101–100 Stone Road West, Guelph, ON, Canada, N1G 5L3

**Keywords:** animal welfare, assurance, dairy cattle, legitimacy, private governance, standards

## Abstract

The governance of farm animal welfare is led, in certain countries and sectors, by industry organisations. The aim of this study was to analyse the legitimacy of industry-led farm animal welfare governance focusing on two examples: the Code of Practice for the Care and Handling of Dairy Cattle and the Animal Care module of the proAction programme in Canada, and the Animal Care module of the Farmers Assuring Responsible Management (FARM) programme in the United States (US). Both are dairy cattle welfare governance programmes led by industry actors who create the standards and audit farms for compliance. We described the normative legitimacy of these systems, based on an input, throughput, and output framework, by performing a document analysis on publicly available information from these organisations’ websites and found that the legitimacy of both systems was enhanced by their commitment to science, the presence of accountability systems to enforce standards, and wide participation by dairy farms. The Canadian system featured more balanced representation, and their standard development process uses a consensus-based model, which bolsters legitimacy compared to the US system. However, the US system was more transparent regarding audit outcomes than the Canadian system. Both systems face challenges to their legitimacy due to heavy industry representation and limited transparency as to how public feedback is addressed in the standards. These Canadian and US dairy industry standards illustrate strengths and weakness of industry-led farm animal welfare governance.

## Introduction

In some countries and sectors, farm animal welfare is governed by a blend of public and private organisations and their associated policies. For instance, farm animal transport, slaughter, and anti-cruelty legislation is governed federally in Canada (Sankoff 2019), and provinces also have anti-cruelty legislation (Fraser *et al.* 2018). In the United States (US), federal public governance of farm animal welfare is mainly for transport and slaughter; individual states have anti-cruelty legislation, and some have farm animal-specific legislation (e.g. Constitution of the State of Florida, Article X, Section 21 bans the use of sow gestation crates in Florida; Mench 2008).

Private organisations are increasingly involved in creating animal welfare standards, creating a shift from ‘government’ having jurisdiction, towards ‘governance’ being shared amongst different organisations (Maciel & Bock 2013). In this paper, we define private governance as *“a form of socio-political steering in which private actors are directly involved in regulating – in the form of standards or more general normative guidance – the behaviour of a distinct group of stakeholders*” (Pattberg 2006; p 591). Private governance organisations typically have little to no involvement with government actors or public infrastructure (Black 2008), develop rules and regulations around an issue without having a specific legal mandate to do so (Neuner 2020), and often arise to fill gaps where public government has failed to address social issues (Rudder 2008). Farm animal welfare is an increasingly politicised issue (Hårstad 2023) of growing societal concern (von Keyserlingk & Weary 2017) that in Canada and the US largely lacks legally mandated standards (Mench 2008; Sankoff 2019). Thus, it is no surprise that farm animal welfare standards have been developed by private actors in these two countries. Examples in Canada and the US include organic standards, animal welfare non-governmental organisation (NGO) standards (i.e. Animal Welfare Approved by A Greener World, https://agreenerworld.org/certifications/animal-welfare-approved/), food retailer standards (i.e. Sprouts Farmers Market, https://www.sprouts.com/about/sustainability/animal-welfare/), and industry-led standards.

Examples of industry-led standards can be seen in the Canadian and US dairy industries, which have taken the lead in creating and enforcing standards for dairy cattle welfare in their respective countries. In Canada, the National Farm Animal Care Council (NFACC), through the initiative of the Dairy Farmers of Canada (DFC), led the creation of the Code of Practice for the Care and Handling of Dairy Cattle (henceforth the ‘Dairy Code’) (NFACC 2023a). Following the requirements set in the Code is mandatory for all dairy farmers in Canada through the Animal Care module of the DFC’s ‘proAction’ programme (DFC n.d.a.). This programme translates requirements from the Code into auditable criteria and implements an on-farm auditing programme (henceforth termed the Code-proAction system).

The NFACC published the first modern version of the Dairy Code in 2009 and updated it in 2023 (NFACC n.d.b.). In 2015, the DFC created an Animal Care module based on the 2009 version of the Dairy Code (DFC n.d.a.), and enforcement was initiated in 2017 (DFC 2018). Codes are reviewed every five years, at which time one of the following recommendations is given: (1) reaffirmation of the Code; (2) an overall update is needed; or (3) specific amendments are needed (NFACC n.d.d.). The aim is for Codes to be updated every 10 years (NFACC n.d.d.).

**Figure 1.**
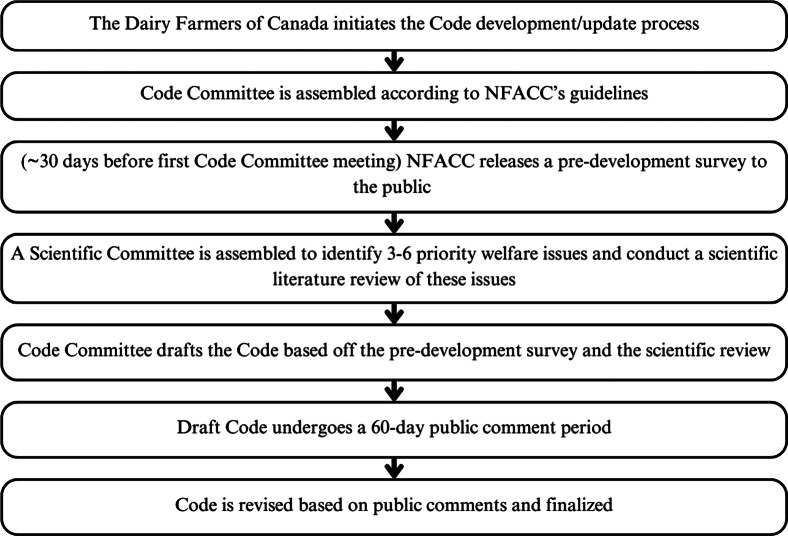
The standard development process for the Code of Practice for the Care and Handling of Dairy Cattle, a Canadian national standard by the National Farm Animal Care Council (NFACC) for dairy cattle welfare.

**Figure 2.**
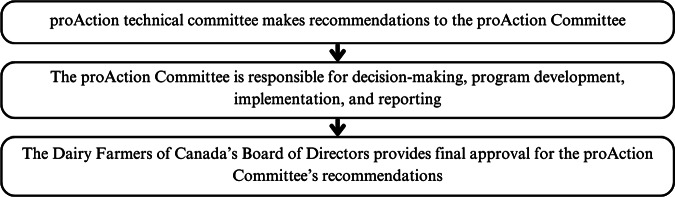
The development process for the proAction Animal Care Module, a set of requirements for dairy cattle care by the Dairy Farmers of Canada.

The Code is developed through the work of a Code Committee and Scientific Committee, with public input through a pre-development survey and public comment period of the draft standard (NFACC n.d.d.) (Figure 1). Specific requirements in the Code are enforced through the DFC’s proAction Animal Care module, which follows NFACC’s Animal Care Assessment Framework. This framework lays out requirements for industry groups when developing assessment programmes based off of the Codes, including guidance on how the Codes are translated into auditable criteria for use on farms (NFACC n.d.g.). The proAction Animal Care module criteria are decided upon by the Animal Care Technical Committee and the proAction Committee, with all recommendations subject to approval by the DFC Board of Directors (DFC 2023b) (Figure 2).

Farms are assessed by proAction auditors (known as validators in the proAction programme, but for the purposes of this paper referred to as auditors) every two years to determine if they have met the Animal Care module’s requirements, with dairy farmers completing a self-declaration on alternate years, of which 5% are audited following their self-declaration (DFC 2023b). As part of the assessment, farms must also undergo cattle assessments where a random sample of cows from each herd is systematically selected according to a sample size calculator and scored for hock, knee, neck, body condition, and locomotion scores against proAction’s requirements (DFC 2023a). Failure to meet proAction requirements may result in the need for a corrective action plan that must be developed and administered within a specified timeline (DFC 2023b).

In the US, the Farmers Assuring Responsible Management (FARM) programme is run by the National Milk Producers Federation (Arlington, Virginia) and Dairy Management Inc (Rosemont, Illinois) (FARM 2024a). In 2022, more than 98% of milk produced in the US was subject to FARM Animal Care standards (FARM n.d.a.). Unlike the Code-proAction system, where standard development and enforcement are managed by separate organisations, the FARM programme develops and enforces their standards. The FARM programme was launched in 2009, and its standards are updated every three years; Version 5 of their animal care programme took effect on July 1, 2024 (FARM 2024a).

**Figure 3.**
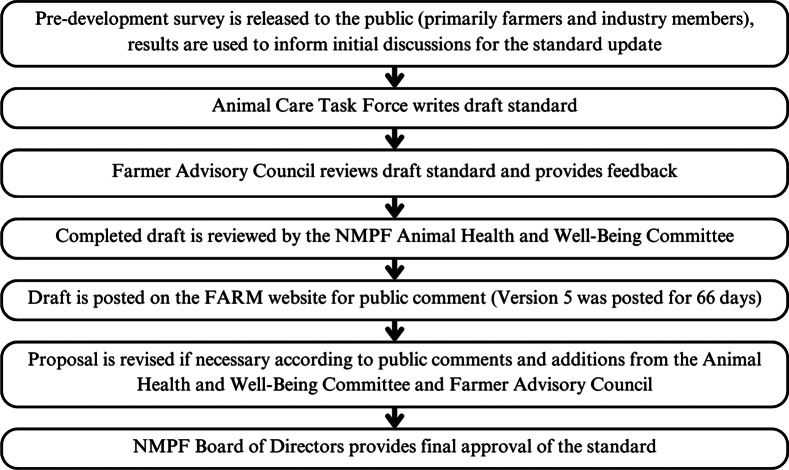
The standard development process for the Farmers Assuring Responsible Management (FARM) programme’s Animal Care standards, a standard for dairy cattle welfare in the United States by the National Milk Producers Federation (NMPF) and Dairy Management Inc.

FARM’s standard is developed through the work of the Animal Care Task Force, Farmer Advisory Council, and NMPF Animal Health and Well-Being Committee, as well as public feedback from a pre-development survey and public comment period (FARM 2023a,b; n.d.a.) (Figure 3).

The FARM programme enforces its Animal Care standards through an on-farm auditing programme, where all farms are audited at least once every three years by a FARM auditor (referred to as evaluators in the FARM programme, but for the purposes of this paper referred to as auditors), and a sample of farms undergo a third-party audit every six months (FARM 2024b). Failure to meet the programme’s standards may result in a corrective action plan with timelines of varying length depending on the requirement that was violated (FARM 2024b).

The Code-proAction system and FARM programme standards are unique compared to other private certification schemes in that they apply to most farmers within the country, thereby functioning as industry-wide standards for animal care. Although adherence is not legally mandated, producers are motivated to comply for a variety of reasons. Suppliers and retailers may require compliance from their producers so they can signal the standard’s attributes to consumers in marketing and labeling schemes (Fuchs *et al.* 2011a), motivating producers to comply for basic market access and better economic returns (Bock & van Huik 2007).

Private governance of animal welfare has been criticised as being less legitimate than public governance, as the former does not need to follow the principles that grant a government legitimacy (Maciel & Bock 2013). Legitimacy can be conceptualised in two ways. Normative legitimacy means that “*the right of an institution to make publicly binding decisions has to be justified by some objective means (e.g. its practices meet a set of standards that* [have] *been agreed upon)*” (Lindgren & Persson 2010; p 451). This approach may be operationalised by assessing whether or not a governing body meets a set of moral criteria, or utilises processes associated with legitimate governance (Schmidt 2020). Empirical legitimacy involves, “[an institution being] *accepted as appropriate and worthy of being obeyed by those affected by its policies*” (Lindgren & Persson 2010; p 451). An institution that establishes a degree of normative legitimacy may not necessarily have empirical legitimacy, and *vice versa.* Some argue that multiple forms of legitimacy are necessary for an organisation to function, as normative legitimacy ensures that an organisation follows legitimate processes and procedures, while empirical legitimacy ensures that the organisation’s policies and goals are complied with and accepted by stakeholders (Hahn & Weidtmann 2016; Kusnezowa & Vang 2021).

Private governance organisations are varied in their structure and goals (Black 2008), and some private organisations may enjoy considerable legitimacy. In some cases, private organisation may be able to create effective outcomes (Maciel & Bock 2013), for example, if they are better resourced with deeper expertise to tackle specific issues (Rudder 2008), or nimbler and thus able to act more quickly compared to governments (Hårstad 2023). In such cases, private organisations may be able to create and enforce effective, specific standards that promote higher welfare than would otherwise be possible.

Criticisms of the legitimacy of private governance often centre around inclusivity, transparency, and accountability. The standard development process of private organisations may exclude the voices of minority groups (Cheyns 2014) or smaller farmers (Fuchs *et al.* 2011a), and consumers may be provided limited opportunity to participate in the standard-setting process (Ryland 2018). Private organisations may also lack accountability for their actions and transparency about their processes (Black 2008). For example, Ryland (2018) argued that the GLOBAL G.A.P. animal welfare standard (https://oc8.globalgap.org/uk_en/) suffers from limited legitimacy due to failure at the point of sale by the retailer to provide information on animal welfare (or improvements thereof) from the certification body. Industry-led private agri-food governance has also been criticised for creating weak standards that are intended to bolster public trust rather than meaningfully addressing the issue at hand (Sharma *et al.* 2010; Moog *et al.* 2015); this criticism may also extend to industry-led animal welfare standards.

As private food industry actors globally push for assurance programmes that meet consumer demands, including those for animal welfare (Fulponi 2006), our aim was to understand potential strengths and weaknesses of private animal welfare governance using the Code-proAction system in Canada and the FARM programme in the US as examples. While Bradley and MacRae (2011) discussed the legitimacy of the NFACC Code development process when it was first introduced, the process has changed since then. To our knowledge, the FARM programme has not been examined through a legitimacy lens. This paper will follow a framework for normative legitimacy, performing a document analysis of publicly available information about dairy cattle welfare standards on the NFACC, proAction, and FARM programme websites.

## Materials and methods

### Normative legitimacy conceptual framework

We have included a reflexivity statement to acknowledge how our contexts may have influenced this research (Olmos-Vega *et al.* 2023). CK is an MSc student in Applied Animal Biology Graduate Programme at the University of British Columbia (UBC) who holds a BA in International Relations from UBC and has been involved in dairy cattle welfare research since 2020. She did not grow up in an agricultural community and has not held a professional role at NFACC, the DFC, or the FARM programme. MvK and DMW are both Professors in UBC’s Animal Welfare Programme. MvK served as a scientific advisor for the FARM programme from 2011–2020 and is now a director at Animal Health Canada, NFACC’s parent organisation. DMW was a founding director of NFACC and currently sits as the scientific representative on the NFACC board. He also co-chaired the scientists’ committee for the development of the 1997 Dairy Code of Practice and was a member of the scientists’ committee for the 2023 Dairy Code of Practice. SMR is a private consultant who actively works with NFACC, DFC, and NMPF to support the development, implementation, and evaluation of the proAction and FARM animal care programmes. None of the listed organisations had any role in the study design, data collection and analysis, decision to publish, or preparation of the manuscript. To mitigate biases, the initial document analysis was completed by CK, who does not have a role at any of the listed organisations. Throughout the analysis process, we intentionally focused upon publicly available information regarding these programmes and held discussions about ensuring that insider information did not influence the direction of the manuscript.

We used an input, throughput, and output framework based on democratic principles to assess normative legitimacy. Koppell (2007; p 190) argues that although it is “*impossible to identify some set of universal demands of legitimacy*” because normative legitimacy criteria vary over “*time, geography, and context*,” in the context of current times, democratic principles are generally seen as universally legitimate across different cultures.

Input legitimacy is dependent upon the ability of a broad range of relevant stakeholders to be involved in decision-making, and that these stakeholders provide meaningful input through their qualifications or expertise (Hahn & Weidtmann 2016). Throughput legitimacy, also termed procedural legitimacy, concerns the accountability, transparency, inclusiveness, and openness to consultation with the public of governance processes (Schmidt 2013; Schmidt & Wood 2019). Output legitimacy is dependent on whether policies are effective at meeting the organisation’s goals and achieving outcomes (Schmidt 2020).

Our input, throughput, and output legitimacy criteria were adapted from work by Kusnezowa and Vang (2021), Schmidt and Wood (2019), and Mena and Palazzo (2012). In total, our framework comprises eight criteria (see Table 1). Using this framework, we performed a document analysis of publicly available materials from the NFACC, proAction, and FARM programme websites. Document analysis is a research method that uses documents as an information resource (Coffey 2012) and can be used in policy research (Karppinen & Moe 2012). Since the Code was updated in March 2023, we refer to this version when discussing the Dairy Code, but in the case of proAction, we refer to the most recently available information, recognising it has not yet been updated to align with the requirements of the 2023 Dairy Code. The fifth version of FARM Animal Care programme standards was published in July 2024 and provides the basis for the information in this analysis.

**Table 1.** Normative legitimacy framework used to analyse the Code-proAction system for dairy cattle welfare governance in Canada, which involves the National Farm Animal Care Council of Canada’s Code of Practice for the Care of Dairy Cattle and the associated proAction Animal Care module by the Dairy Farmers of Canada, in comparison to the US-based Farmers Assuring Responsible Management Programme’s Animal Care module. Legitimacy criteria are categorised according to input, throughput, and output legitimacy

Legitimacy criteria	Type of legitimacy
Stakeholder representation (Kusnezowa & Vang 2021) Standard-setting organisations should include a balanced range of stakeholders that are affected by the issue. Including high profile representatives (i.e. known authorities whose word already holds a high level of respect) may help to bolster this form of legitimacy as well.	Input
Using and referring to expertise (Kusnezowa & Vang 2021) Using and referring to expertise can bolster input legitimacy because experts can provide subject-matter knowledge that may increase the quality of the standard. Specifically, we focus on the incorporation of science into the standard development process.	Input
Transparency (Kusnezowa & Vang 2021) We follow the definition of transparency by Fuchs *et al.* (2011a; p 357) as, “*the provision of timely, reliable and comprehensible information on the governance and performance characteristics of the standards.*” We focus on transparency of the organisation’s activities and audit outcomes.	Throughput
Accountability (Schmidt & Wood 2019) This criterion refers to the extent to which these organisations and participating farms are held accountable for their decisions and face consequences for not meeting claims.	Throughput
Facilitating participation (Kusnezowa & Vang 2021) This criterion refers to the extent to which different stakeholder groups are provided with the opportunity for equal participation in the standard development process. Though these authors focused on the possibility of language barriers and technical jargon that bars participation from select groups, we will also focus on the accessibility of the public comment period.	Throughput
Consensus decision-making (Kusnezowa & Vang 2021) Under consensus decision-making, all stakeholders should reach an agreement and consent to a decision that they are making. Although complete agreement on decisions is often not possible, and attempting to force complete agreement may be democratically counterproductive (Martí 2017), Kusnezowa and Vang (2021) note that it is important for stakeholders to focus on areas of agreement during the decision-making process and aim to reach a decision that all stakeholders can accept. This form of decision-making can also act as a preventative measure against more powerful stakeholders being able to sway decisions (Biermann & Gupta 2011).	Throughput
Participation in the standard (Mena & Palazzo 2012) This criterion refers to the number of actors that are bound to compliance with a standard (Mena & Palazzo 2012). According to Bernstein and Cashore (2007), broad participation in a private standard is essential for its legitimacy because it can help to set norms for the industry.	Output
Efficacy of the standard (Mena & Palazzo 2012) This criterion refers to the extent to which the standard provides an effective solution to the issue it aims to address. Efficacy might be evaluated in different ways, including the extent to which the standard improves public trust in the industry and its practices, or the extent to which the standard improves the welfare of animals. In our analysis, we focus on the latter.	Output

Webpages and documents were retrieved from September 2023 to December 2023 by searching each organisations’ websites including annual reports, videos, webpages explaining their standards and processes, webpages detailing the committees and members involved in the organisation, and the manuals associated with each set of standards that describe in detail the programme’s requirements. This search was supplemented by additional searches from February 2024 to April 2024 and in September 2024 to include newly released information on FARM Animal Care version 5.

Following the searches, materials were downloaded to Zotero, a citation manager software. All of the retrieved materials were carefully read, and materials relevant to our legitimacy framework were deductively analysed (12 from the NFACC website; five from the proAction website; 12 from the FARM website) using manual thematic coding (Guest *et al.* 2012), where pieces of text that corresponded to the legitimacy criteria in our framework were highlighted and noted in the ‘notes’ section of Zotero.

## Results

### Stakeholder representation

The Code-proAction system and the FARM programme both categorise representation in terms of different organisations and interests. The NFACC 2023 Dairy Code included the following Code Development Committee membership: six dairy farmers, one veterinarian, one animal welfare organisation representative, two provincial government representatives, one processor, two researchers (who were also on the Scientific Committee), three programme implementation experts, one technical expert, two federal government representatives, two allied industry (beef cattle and veal) representatives, and one industry liaison (a representative from the DFC) (NFACC 2023a).

The Animal Care Technical Committee responsible for developing the proAction Animal Care module includes 13 members with representation from farmers, scientists, veterinarians, and industry specialists (DFC 2023b). Representatives are chosen via nominations by the commodity association (in this case, the DFC) (NFACC n.d.g.). The Animal Care Technical Committee reports to the proAction Committee, where 15 of the 16 voting members are dairy farmers (DFC 2023b). Final approval of proAction standards is the sole responsibility of the DFC’s Board of Directors (DFC 2023b). Of note is that six of the 19 committee members were dairy farmers and one of the 19 committee members was a representative from a dairy organisation in the NFACC standard development process; other groups were less well represented (e.g. one animal welfare organisation representative). The proAction Animal Care committee also features heavy representation from dairy farmers and associated staff.

FARM programme’s committees involved with the development of Version 5 of the Animal Care Module include the Farmer Advisory Council (100% farmers), the Animal Care Task Force (26% farmers), and the Animal Health & Well-Being Committee (60% farmers) (FARM 2023b). Final approval of the FARM Animal Care standards is under the purview of the NMPF Board of Directors, of which 65% are farmers (FARM 2023b). For each of these bodies, non-dairy-farmer membership is comprised of veterinarians, animal scientists, milk processors, and dairy co-operative representatives (FARM 2024a). In the US, many dairy farms supply milk to a dairy co-operative (United States Department of Agriculture [USDA] 2005); thus, dairy co-operatives have a stronger presence in the FARM programme compared to Canada where dairy farms operate independently but supply milk jointly to the milk pool of their region following the supply management system (Government of Canada 2023).

We have discussed representation on the different committees in terms of membership that speaks directly to dairy farming interests (i.e. dairy farmers) vs others (i.e. veterinarians, government representatives, etc), but we acknowledge that the interests of individual representatives may not always be clear. Individuals that are not direct representatives of dairy farming interests may still be beholden to the industry’s interests; for example, veterinarians may be financially dependent upon dairy farmers who are their clientele, and researchers may be beholden to organisations like the DFC who fund their research.

Both programmes also attempt to include the voices of individuals and organisations who are not part of a decision-making committee through a survey administered prior to standard development and via a public comment period when the draft standard is released. For example, the most recent Dairy Code pre-development survey reported that they primarily received responses from the following categories: ‘general public’ (43.8% of respondents), ‘consumer’ (39.1% of respondents), ‘animal welfare advocate’ (36.7% of respondents), and ‘dairy producers’ (20.8% of respondents) (respondents were able to select more than one category) (NFACC 2019). The primary responses to the public comment period were divided as follows: dairy producers (40%); consumers (17%); and what they termed ‘concerned citizen/animal welfare advocate’ (31%) (NFACC 2023b).

In contrast, the FARM programme’s pre-development survey primarily received responses from industry-affiliated personnel: 63% dairy farmers; 16% dairy processing organisation staff; and 12% veterinarians (Roche *et al.* 2022). Only 1.5% of respondents represented the public and consumers (Roche *et al.* 2022). The specific respondent demographics for the public comment period were not publicly shared, but included individuals, co-ops, and industry allies (FARM 2022).

### Using and referring to expertise

Both the Code-proAction system and the FARM programme state their commitment to science-based animal welfare standards. The FARM programme was certified through the International Organisation for Standardisation (ISO) Technical Specification during Version 4 (V4), which certifies that V4 met international animal care standards (no public information was available regarding the latest version [V5; FARM 2020]).

Although the Dairy Code and FARM programme standards are based upon science, animal welfare is a value-laden science (Fraser et al. 1977), meaning that organisational values and the values of representatives sitting on committees influence the content and enforcement of standards. Organisational definitions of animal welfare appear to avoid value-laden language, focusing on the animal’s state of being and outcomes. NFACC defines animal welfare according to an animal’s ability to physically, physiologically, and psychologically cope with its conditions (NFACC n.d.a.) while the FARM programme defines animal welfare as the outcomes experienced by the animals, which can be influenced by “*housing environments and facilities, management practices, standard operating procedures or protocols, and direct human-animal interactions and handling*” (FARM 2024a; p 5). The Dairy Code attempts to reflect the scientific consensus in its peer-reviewed Scientific Report (by DeVries *et al.* 2020), which summarises the scientific literature about the pre-defined priority issues, and how these studies were designed, conducted and interpreted. However, both this review and the original studies included in the review may also be subject to biases, including the interests and values of the researchers and the funders of research.

### Transparency

The NFACC publicly releases yearly progress reports (NFACC n.d.e.), information about the Code development process (NFACC n.d.d.), and related documents (e.g. the Scientific Report by DeVries *et al.* 2020). ProAction publishes yearly progress reports (DFC n.d.b.) and documents with information regarding their standard requirements and enforcement policies (DFC 2023b). Both organisations are also transparent about the individuals involved in the standard development process and identifies them by name (DFC 2023b; FARM 2024a).

The FARM programme publishes documents online concerning the organisation’s activities, including annual reports since 2014 (FARM n.d.b.), information on governance structure and membership (FARM 2024b), Industry Town Hall recordings that discuss standard development progress (FARM n.d.a.), and survey results (Roche *et al.* 2022). The FARM programme is also transparent about the individuals involved and identifies them by name (FARM 2024a). Timeliness of information provision is one component of transparency (Fuchs *et al.* 2011a) where the FARM programme differs from the Dairy Code, in that updates are published regularly throughout the development process of the standard for the FARM programme (FARM n.d.a.).

Transparency relating to the public comments received is limited in both systems. The NFACC released a summary of comments received for the 2023 Dairy Code (NFACC 2023b), and the FARM programme provided a summary of public comments through Industry Town Hall video livestreams about the standard development process (FARM n.d.a.) and a written document (FARM 2023a), but neither organisation published individual comments.

Transparency of outcome measures and the results of on-farm visits vary between the systems. ProAction released limited information about on-farm audits conducted in 2017, including that 88% of farmers had an SOP for calf feeding and that 90% of sampled cattle fell within the acceptable range for locomotion scoring (DFC 2018). However, to our knowledge, the proAction Animal Care module has not publicly released audit outcomes since 2017. In contrast, the FARM programme has published selected outcome measures annually since 2016 (FARM 2016). In their 2022 annual report, FARM released information on outcome measures relating to the number of evaluations performed, percentages of farms required to correct certain types of infractions, and percentages of farms that met requirements to have a valid, signed Veterinarian-Client-Patient relationship form and stockmanship training requirements (FARM 2022).

### Accountability

ProAction and the FARM programme both require on-farm audits of participating dairy farms to hold them accountable to their standards. Under proAction, failure to meet certain requirements results in a major or minor non-conformance, which must be remedied through a corrective action plan and within a timeline made in collaboration with a dairy professional (i.e. the herd veterinarian) (DFC 2023a,b). Loss of licencing can occur if major issues are unresolved within the specified timeline (DFC 2023a). Failure to meet other requirements results in demerits (DFC 2023b). Farms may have demerits but still retain licencing; proAction states that this “*allow[s] farmers to have some flexibility and promote continuous improvement*” (DFC 2023b; p 5). However, our review of the proAction Animal Care documents did not reveal the threshold for demerits, or if demerits require corrective action. Animal abuse cases uncovered by proAction are reported to the authorities associated with animal abuse regulations (DFC 2023b).

The FARM programme also enforces its standards through an on-farm auditing programme. Farms failing to meet certain FARM standards may be required to develop, depending on the infraction, an Immediate Action Plan, a Mandatory Corrective Action Plan, or a Continuous Improvement Plan (FARM 2024b). These plans differ in terms of the length of time allowed for the farm to resolve the infraction; resolution times are immediate, within nine months, or within three years, respectively. Failure to comply can result in a Conditional Decertification that will block the farmer from selling milk under FARM’s certification until the issue is resolved (FARM 2024b). In addition, FARM has a Willful Mistreatment or Neglect Protocol that is triggered in response to allegations of animal abuse; under this protocol, farms may be inspected by a third party and decertified based on the outcome of that inspection (FARM 2024b). Facilities may apply for reinstatement but must pass additional audits (FARM n.d.c.). Our review of FARM documents found no data on the frequency of this outcome.

An important feature of accountability is the extent to which non-compliant farms are faced with consequences. Our review of both systems shows that farmers can face a tangible penalty (loss of licencing or access to their milk market) in both the US and Canada for failing to meet certain standards. However, limited transparency regarding the audit outcomes (i.e. how many farms fail and why) in both systems limits accountability and makes it unclear the degree to which non-compliant farms face these consequences. It is also unclear if corrective actions and their timelines are appropriately matched to requirements.

The independence of the auditor is crucial to a valid audit (Tepalagul & Lin 2015). According to the ISO/IEC (2020), a second-party audit is “*performed by a person or organization that has a user interest in the object of conformity assessment*” and a third-party audit is “*performed by a person or organization that is independent of the provider of the object of conformity assessment and has no user interest in the object.*” Third-party audits are typically considered more legitimate than second-party audits given the auditor’s greater degree of independence (Mena & Palazzo 2012). The proAction farm visits that are conducted by a proAction-trained auditor qualify as a second-party audit. The cattle assessments conducted under proAction are performed by Holstein Canada, an organisation that performs purebred Holstein conformation evaluations. This organisation might be considered third-party but may be considered more accurately a second-party audit in some cases as the Holstein Canada auditor may have an established relationship with the farm through conformation evaluations. FARM audits are performed by a FARM-trained second-party auditor such as a dairy co-operative representative, but a sample of farms undergo a third-party audit by a contracted independent livestock auditing company to assess agreement between the second- and third-party auditors (FARM 2024b).

### Facilitating participation

Many of the individuals included in the decision-making processes in the Code-proAction system and the FARM programme have knowledge about the dairy industry and thus would likely understand the issues and standards. However, Bradley and Macrae (2011) note that food retailer and government representatives in the Code development process may lack the knowledge to provide meaningful input. This critique may also apply to food retailer and processor representatives in the FARM programme. Members of the public who are not involved with dairy farming may also find it difficult to understand the specifics of these standards when engaging with the pre-development surveys and public comment periods.

In analysing the accessibility of the public comment period, we looked for information regarding how the public comment period is disseminated. The NFACC encourages industry and non-industry members of the public to submit comments (NFACC n.d.f.), and notes that industry organisations typically share the link within their networks (NFACC n.d.h.). It is unclear how widely the link is distributed to other stakeholder groups, though we were able to find evidence that one animal welfare organisation (the British Columbia Society for the Prevention of Cruelty to Animals) shared news articles about the Dairy Code (BC SPCA 2022). A total of 45,470 comments from 5,884 respondents and 50 organisations across different stakeholder groups were received for the 2023 Dairy Code (NFACC n.d.c.); the volume of comments across different stakeholder groups indicates a degree of success in making the public comment period accessible.

The FARM programme’s public comment period is announced on various online channels, and the most recent comment period for the FARM programme’s V5 collected 308 comments (FARM 2022). It is not clear why the Code received almost 20-times as many comments as the FARM program when Canada has approximately one-tenth the population of the US, but we hypothesise that this difference may be due to differences in how the public comment period is announced and disseminated.

### Consensus decision-making

The NFACC emphasises their commitment to using a consensus decision-making model, meaning that all stakeholders are committed to making decisions that all representatives find acceptable and consent to the final decisions made (NFACC n.d.a.). The deliberations during the Code development process are not public so it is unknown how this consensus decision-making model is used, but NFACC considers it to be a keystone of their process.

Based on publicly available documents, it is unclear how proAction development committees and the FARM programmes’ committees make decisions and whether they follow a consensus-based model.

### Participation in the standard

Given that 98% of milk produced in the US is from a FARM-certified farm (FARM n.d.a.), and all dairy farmers in Canada are bound to proAction to maintain market access (DFC n.d.a.), both standards have high participation.

### Efficacy of the standard

**Figure 4.**
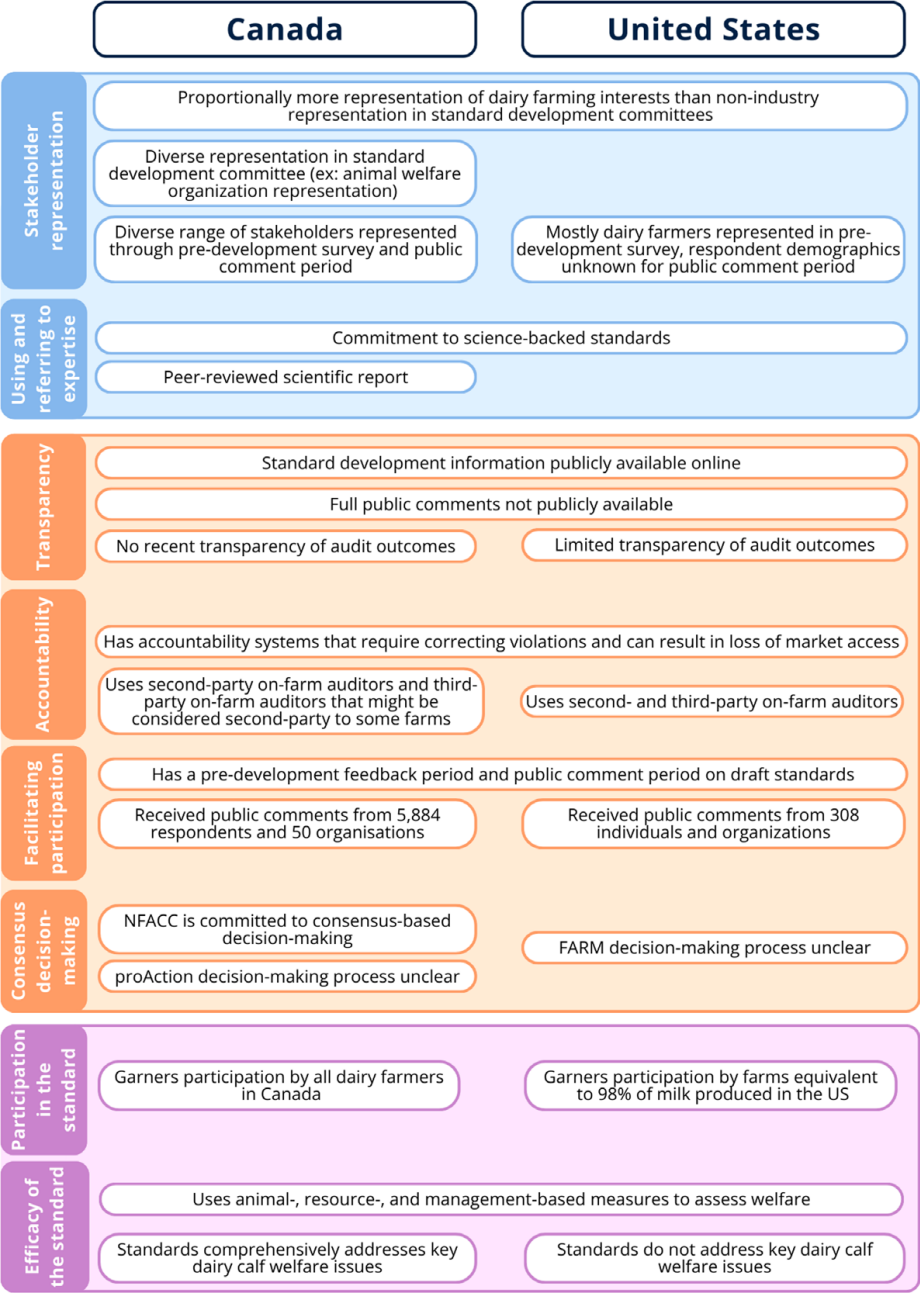
Synthesis of key results when comparing the Canadian National Farm Animal Care Council (NFACC) Code of Practice for the Care and Handling of Dairy Cattle and associated proAction programme (‘Code-proAction system’) to the Farmers for the Assurance of Responsible Management (FARM) programme in the United States (US) according to a normative legitimacy framework.

Efficacy of a standard in ensuring positive animal welfare is made of multiple components, including which animal welfare issues the standard addresses, and the quality of measurements used to assess animal welfare. Given that animal welfare is value-laden and different people will have different definitions of good animal welfare (Fraser *et al.* 1997), it is difficult to define which animal welfare outcomes would constitute an effective standard. However, at a baseline, the standard should address key animal welfare issues that are well accepted based on the available scientific literature. Although an in-depth analysis of the animal welfare issues covered in the Code-proAction system and the FARM programme is beyond the scope of this paper, we have selected three well-researched areas of dairy calf welfare covered in a review by Costa *et al.* (2019) as a way of illustrating where standards set out by both programmes sit in relation to the available welfare science knowledge (Table 2). This analysis found that the Code-proAction system addresses calf welfare issues to a greater degree than the FARM programme (see Figure 4), but we advise caution in drawing conclusions from this evidence until other Animal Care modules are subject to the same level of assessment.

**Table 2.** A comparison of requirements in the Canadian National Farm Animal Care Council (NFACC) Code of Practice for the Care and Handling of Dairy Cattle and the associated proAction Animal Care module (‘Code-proAction system’), and version 5 of the Farmers Assuring Responsible Management (FARM) programme in the United States, to a review by Costa et al. (2019) of dairy calf welfare issues

Dairy calf welfare issue (Costa *et al.* 2019)	Code-proAction system requirement	FARM programme V5 requirement
Social (rather than individual) housing of pre-weaned dairy calves has been shown to be important for their welfare by allowing the expression of natural social behaviours and promoting a more positive affective state	The 2023 Code requires social housing for indoor-housed calves by 4 weeks of age (this requirement will not be enforced until 2031 and has not yet been added to proAction), outdoor-housed calves will be excluded from this requirement (NFACC 2023a)	Social housing is not a requirement
Restricted milk feeding (under 15% of body weight) causes behaviours associated with hunger and reduced growth	proAction requires feeding calves 20% of their body weight for the first month (DFC 2023b)	Requires pre-weaned calves to receive “*a volume and quality of milk or milk replacer to maintain health, growth, and vigor until weaned or marketed*” (FARM 2024a; pp 95). Volume is not specified in the requirement.
Dehorning is a painful procedure and pain control should include a local anaesthetic and analgesic	Requires the use of anaesthetic and analgesic prior to disbudding (DFC 2023b)	Requires pain mitigation during disbudding. Recommends the use of a local anaesthetic and systemic pain relief but does not require the use of both (FARM 2024a)

Another component of efficacy is the quality of measurements used to assess animal welfare. Fraser and Koralesky (2017) summarise three ways that animal welfare can be measured in animal welfare standards: animal-based, where animal measurements are used (i.e. acceptable body condition score); resource-based, where requirements for the environment are specified (i.e. minimum space allowances); and management-based requirements, which require that certain practices should be carried out (i.e. using pain control when disbudding calves). According to these authors, different types of requirements are more applicable in different situations and achieve different outcomes, but animal-based measurements are often preferred because they can be used in a variety of settings and directly assess animal welfare.

NFACC requires that animal care assessment programmes, such as the DFC’s proAction, use all three types of assessment measures and include target levels when possible (NFACC n.d.g.). Indeed, proAction’s Animal Care standards use a combination of these measures; for example, by doing locomotion scoring and setting target levels through the cattle assessments (animal-based), requiring bedding for all animals (resource-based) and requiring pain control when disbudding calves (management-based) (DFC 2023b). The FARM programme also uses all three types of assessment measures; for example, by doing body condition scoring and setting target levels (animal-based), requiring access to water (resource-based), and requiring non-ambulatory animals to be moved in a specific way (management-based) (FARM 2024a).

A feature of both systems is that evaluation of many of the management-based requirements are tied to an evaluation of written protocols. For example, the proAction programme requires that a written protocol be established for calf feeding, and the reference manual states that auditors may interview farm staff about the content of the written protocol to ensure that practices are followed on-farm (DFC 2023b). Many FARM requirements are tied to a written protocol as well, such as requiring a written protocol for non-ambulatory cattle management (FARM 2024a). Depending on the requirement, the FARM auditor may read the protocol, verify that the protocol reflects on-farm practices by interviewing the owner or staff, observe on-farm practices, or carry out a combination of these practices (FARM 2024a). For example, access to water requires a written protocol, and may also be assessed via interview, observation, or both (FARM 2024a).

## Discussion

This paper aimed to analyse the Code-proAction system and the FARM programme according to a normative legitimacy framework to understand the strengths and weaknesses of industry-led private animal welfare governance models. In terms of input legitimacy, both systems receive input from a range of stakeholders through direct representation in decision-making committees and public feedback, and are informed by research from the natural sciences. The FARM programme’s decision-making committees mainly represent dairy farmers and industry interests; whereas the Code process includes more representation from non-industry perspectives, such as government and an animal welfare organisation. In terms of throughput legitimacy, both systems are transparent about organisational information, and both have accountability systems in place intended to ensure that dairy farmers meet the requirements in the standard. The FARM programme provides more transparency into audit outcomes than proAction. The systems differ in their internal decision-making model; a strength of NFACC is that it is committed to a consensus-based model while the decision-making models for proAction and the FARM programme are not publicly shared. In terms of output legitimacy, both programmes enjoy wide participation from across their respective dairy industries and use a combination of ways to measure animal welfare, some of which are tied to written protocols. When only looking at three calf welfare priorities, the Code-proAction system appears to have clearer requirements that are more effective at ensuring calf welfare compared to the FARM programme.

A key finding of this paper is that in both systems the majority of the standard development committee seats are held by dairy farmer representatives. We recognise that within the Canadian system there is a more diverse range of stakeholders on the Code Committee, and the potential bias associated with having a higher number of dairy farmer seats might be mitigated by the use of a consensus decision-making model. However, this finding raises a question as to how representation should be distributed among dairy farmers and other stakeholder groups without compromising the legitimacy of the standard development process. One mechanism through which representation may be balanced is to reconfigure the representation of interests and distribution of decision-making power in the standard development committees. The Forest Stewardship Council (FSC), an international forest sustainability certification organisation, has attempted to resolve issues with balanced representation by requiring consensus between three voting chambers (economic, social, and environmental), thereby balancing the interests of different stakeholders (FSC 2022). Exploring more balanced ways of representing interests during decision-making processes may bolster legitimacy.

However, balancing representation must be carried out with empirical legitimacy in mind. Meeting a criterion of normative legitimacy, such as balancing representation, does not guarantee empirical legitimacy; the latter being crucial because it impacts how and if stakeholders comply or agree with policies (Bradley & MacRae 2011). Cashore (2002; p 511) identifies different audiences for legitimacy, with a Tier I audience defined as those “*that have a direct interest in the policies and procedures of the organizations*” and may also be conceptualised following Elliott (2012; p 375) as, “*those who are most directly affected by rules*” whose perceived legitimacy is important because, “*they are required to implement them or to conform them.*” In this case, the perceived legitimacy of dairy farmers and co-operatives in both countries might be considered most important. Indeed, it is important that actors who have direct influence over the lives of the cattle should be involved in the standard development process to ensure that the standards are meaningful and manageable (Sharma *et al.* 2010) and that requirements garner enough acceptance to become a social and cultural norm that invites compliance even without stringent surveillance (Bernstein & Cashore 2007).

Existing research suggests that dairy farmers have mixed perceptions of proAction and the FARM programme. Focus groups with dairy farmers in Canada found that some praised proAction for being industry-driven and thought the programme was beneficial because it provided consistency, fostered collaboration, and improved public perceptions of the dairy industry (Ritter *et al.* 2020), but Ida *et al.* (2023) found that some farmers were dissatisfied with certain proAction record-keeping requirements. The FARM programme appears to face mixed perceptions as well (Rink *et al.* 2019). In a survey of 487 farmers, 83.3% stated that more farmer input was necessary, indicating a lack of perceived input legitimacy. The strong presence of dairy co-operatives rather than individual farmers in the FARM programme might explain why dairy farmers had low perceived input legitimacy of the programme. Perceived output legitimacy appears mixed; 45.6% of farmers reported that they thought the programme did not have value, 47.4% of farmers reported that they did not think the programme benefitted cattle health or well-being, and 46.4% of farmers indicated that the FARM programme addressed consumer concerns (Rink *et al.* 2019). Based on this evidence, there is the risk that balancing representation to include more non-dairy farming representation may reduce perceived legitimacy by farmers.

Cashore (2002; p 511) defined a Tier II audience as those “*within civil society that have a less direct but equally important role in granting legitimacy.*” In the current examples, this would include citizens and consumers, key stakeholders whose perception of legitimacy is crucial to the social sustainability of the dairy industry (von Keyserlingk & Weary 2017). The available evidence suggests that public trust in industry-led animal welfare governance is low. Spain *et al.* (2018) reported that US consumers found animal welfare legislation and private third-party certifications to be more trustworthy than private industry initiatives because they were perceived to be more objective. Similarly, Uzea *et al.* (2011) surveyed Canadian members of the public and reported that government verification of animal welfare was considered more trustworthy compared to verification by farmers, processors, third-party actors or supermarkets. Thus, the factors that affect perceived legitimacy likely differ for farmers and non-farmer participants; farmers may prefer more farmer input into the standard development process while citizens might prefer less dairy industry input. We conclude that balancing representation to include more non-dairy industry representation such as animal welfare researchers and non-industry organisations may be a step towards improving citizen perceptions of legitimacy, but that it is important that standard development committees meet dairy farmers’ needs by considering the feasibility of required standards.

Although the Code-proAction system and FARM programme appoint representatives on decision-making committees with the expectation that they speak for their stakeholder group’s interests, there is no guarantee that this occurs. Using an example from a social responsibility standard (i.e. ISO 26000), Hahn and Weidtmann (2016) discuss how NGOs are often heterogenous, resulting in difficulties when selecting a representative. These authors go on to state that whilst further dividing the NGO stakeholder group into smaller subgroups may be more representative of differing NGO interests, this approach may also lead to practical difficulties associated with efficient decision-making. Although increasing the number of representatives within each stakeholder group may be impractical, we recommend intentional efforts to appoint a diverse group of representatives within stakeholder groups to mitigate concerns that the needs of minority groups or smaller farmers might go unrepresented (Fuchs *et al.* 2011a; Cheyns 2014). For example, a diverse panel of dairy farmer representatives might include those with varying farm sizes, gender identities, farming styles, and values towards animal welfare. We also recommend efforts to ensure that diverse representatives are able to participate in the standard development process given that those from underrepresented groups may require accommodations to participate fully.

Some concerns regarding diverse representation might be addressed through better use of the public comment period. A public comment period can function as a public participation tool by providing a forum for the various publics to provide input into the standard while also capturing a broad range of perspectives. Arnstein (2019) differentiates between: (1) non-participation, where authorities use public participation to influence the public rather than meaningfully involving them in decision-making or considering their voices; (2) tokenistic participation, where the public’s voice is heard and considered in a top-down fashion by authorities but may not be able to influence decision-making; and (3) public power, where the public is able to influence decision-making through shared power with authorities or complete power over governance. The public comment approach currently used by the Code or the FARM programme might be considered somewhere between non-participation and tokenistic participation.

Although lower forms of participation are seen as less valuable in Arnstein’s model, others suggest that the appropriate level of participation may be context dependent, and that ‘lower’ forms of participation are not inherently bad (Thomas 1990). In the context of the Code-proAction system and the FARM programme, where there is a need to balance broad representation and public input with practicality of implementation, one possible step forward is to expand already existing public feedback periods in ways that increase transparency. For instance, in both the Canadian and US systems, the individual public comments are summarised but not published as individual comments. In contrast, comments from the European Food Safety Association’s (EFSA) public comment period are published online and the EFSA publicly justifies why they have accepted or rejected suggestions (Finardi *et al.* 2012).

Increasing input legitimacy through increased public participation has been criticised for resulting in reduced output legitimacy in the form of less effective policy outcomes (Kruuse *et al.* 2019). Proponents of private governance tend to emphasise the benefits of improved efficacy and hence output legitimacy (e.g. better policies are made which are more quickly adopted) as a worthy trade-off for reduced input legitimacy (Henson 2011). However, Lindgrenn and Persson (2010) found that, within the context of EU chemicals policy, increased perceived input legitimacy increased perceived output legitimacy. Although they concluded that more empirical research is needed as the relationship between input and output legitimacy may be context-dependent, this finding highlights the potentially complementary nature of input and output legitimacy and benefits to animal welfare standard development.

Whether increased input legitimacy limits or enhances output legitimacy may be dependent, in part, on how able an organisation is to read, process, and respond to the feedback it receives – organisations that lack the resources to properly do so may face limited output legitimacy. The almost 400-fold increase in public comments from the 2009 to the 2023 version of the Dairy Code illustrates the practical difficulties that can be associated with managing a public comment period, in addition to the taxing nature of reading through sometimes highly critical comments (Spooner 2017). To increase legitimacy, organisations are encouraged to develop methods to effectively manage public feedback.

Another key finding is that transparency of animal welfare outcomes is nearly non-existent in the Code-proAction system and limited for the FARM programme. Failure to provide publicly facing transparent data on outcomes may be seen as preventing these organisations from being held accountable for their actions, as it is difficult to if standards are being meaningfully enforced. A lack of transparency can also negatively impact public perceptions of farm animal welfare and trust in farmers (Robbins *et al.* 2016). Walker and von Keyserlingk (2018) argued that farms should undergo a first- or second-party audit to initiate confidential discussions about their farms’ performance and be provided with anonymous reports benchmarking their performance against other participating farms, a step that could better prepare them for the more objective third-party audit. Afterwards, publicly releasing aggregated, anonymous data from the third-party audit would bolster transparency.

Our analysis also found that both systems use written protocols as part of the assessment of management-based requirements. Although both systems have measures in place to check if the written protocol is followed on-farm, the presence of an SOP does not guarantee that the farm follows the practices. Mills *et al.* (2020) found that some farmers make standard operating protocols (SOPs) only to satisfy proAction requirements, and do not refer to these in practice. This finding suggests that written protocols may not be an effective way to evaluate animal welfare, and the efficacy of including them as an item in an animal welfare standard may be dependent on the training and thoroughness of the auditor. However, a potentially imperfect reflection of the state of care provided to the animals on a farm is an issue that many animal welfare audits face and must continue to grapple with (Webster 2005), and is not unique to proAction or the FARM programme.

Overall, we argue that private organisations should strive for both normative and empirical legitimacy should they wish to establish themselves as rightful authorities and rule-makers for animal welfare. Broadly speaking, having legitimacy can support the stability of an organisation and its activities, and can enhance people’s compliance with, acceptance of, and trust in said organisation (Suchman 1995). Further, legitimacy also allows an organisation to establish authority over its claimed jurisdiction (Black 2008) and can be used to justify its right to rule-making over competitors (Smith & Fischlein 2010).

Agri-food industry self-regulation of food safety, environmental concerns and animal welfare have been criticised for serving industry interests (Fuchs *et al.* 2011a), highlighting the need for industry organisations to justify their legitimacy as decision-makers. Sharma *et al.* (2010) argues for industry self-regulation to be considered legitimate, it must be done in good faith and address social issues. Otherwise, industry-led efforts may simply reflect regulatory capture. Lacy-Nichols and Williams (2021) criticise food corporations for posing as “part of the solution” by funding public health campaigns and co-opting regulatory efforts as a protective measure to avoid more substantial changes within the industry, while continuing to promote the consumption of unhealthy foods. If animal welfare standards are to be created by industry organisations, such as with the Code-proAction and FARM programmes, then legitimacy is crucial for their acceptance.

Lastly, it is important to acknowledge the changing character of legitimacy. Although some scholars conceptualise legitimacy as a static state that can be achieved when a series of set criteria are met (Suddaby *et al.* 2017), legitimacy may also be conceptualised as a social process through which standards become a shared cultural belief (Johnson *et al.* 2006). In this way, legitimacy is “*actively and continually negotiated*,” and because it interplays with social conditions, can have varied characteristics across different contexts (Suddaby *et al.* 2017; p 459). The results of the current study should be viewed within the context that we evaluated these systems at one point in time, using one set of normative criteria.

## Study limitations

This analysis relies upon publicly available documents from the NFACC, proAction, and FARM websites; some aspects of how these organisations govern themselves may be hidden from the public such that the information gathered from these websites may not reflect the actual workings of each programme.

Normative legitimacy frameworks and concepts arose from research on governments; it can be argued that these frameworks may not be the most appropriate for evaluating private governance (Henson 2011). Hahn and Weidtmann (2016) note that elections are traditionally posed as a legitimacy granting feature for governments that are typically not present in private governance. Although input, throughput, and output legitimacy have been developed to represent the legitimacy of governments, they have also been used to evaluate the legitimacy of private governance organisations (i.e. analysis of the ISO/CEN Standard for Sustainable and Traceable Cocoa by Kusnezowa & Vang 2021; analysis of the NFACC Codes of Practice by Bradley & MacRae 2011) and provided the best fit for our analysis.

Our evaluation of output legitimacy is limited by a lack of comprehensive analysis of the extent to which these programmes address important animal welfare issues. We encourage future research to undertake a more comprehensive analysis of the efficacy of these programmes.

### Animal welfare implications

Animal welfare is impacted by a variety of factors, including governance and policies that set standards for their care. As private governance of animal welfare becomes more widespread, the results of this research highlight how private governance systems might be strengthened to improve their legitimacy using the Canadian Code-proAction system and US FARM programme as examples. Improving the legitimacy of these standards also has the potential to improve the welfare of dairy cattle under these systems by increasing the breadth of animal welfare concerns represented during standard development, improving compliance, and upholding more transparent enforcement of standards.

## Conclusion

The Canadian Code-proAction system and US FARM programme are two examples of private industry-led governance of animal welfare. Our study contributes to the growing literature on private agri-food governance (Fuchs *et al.* 2011b), and expands this body of literature beyond discussions of food and sustainability governance to include animal welfare governance. As governance of farm animal welfare in some jurisdictions continues to shift into the private realm (Maciel & Bock 2013), our findings illustrate the strengths and weaknesses of such systems according to a legitimacy framework.

Based on our findings, we suggest that the Code-proAction system and the FARM Programme would benefit from continuing to build democratic characteristics into their organisations and standard development processes. This could be achieved by better balancing interests on standard development committees, and improving citizen perceptions of legitimacy, though care must be taken to ensure that standards remain feasible for dairy farmers. In addition, both organisations would benefit from additional transparency regarding public comments and animal welfare outcomes from audits.

We recommend future research to examine other forms of animal welfare governance through a legitimacy lens to identify their strengths and weaknesses. We also recommend future empirical research on the legitimacy of animal welfare governance to identify which democratic characteristics might be considered as important to different stakeholder groups. Lastly, given that proponents of private governance often claim it has more effective outcomes (Henson 2011), future research should investigate this claim in terms of farm animal welfare governance.

## References

[r1] Arnstein SR 2019 A ladder of citizen participation. Journal of the American Planning Association 85: 24–34. 10.1080/01944363.2018.1559388

[r3] Bernstein S and Cashore B 2007 Can non‐state global governance be legitimate? An analytical framework. Regulation & Governance 1: 347–371. 10.1111/j.1748-5991.2007.00021.x

[r92] Biermann F and Gupta A 2011 Accountability and legitimacy in earth system governance: A research framework. Ecological Economics 11: 1856–1864. 10.1016/j.ecolecon.2011.04.008

[r4] Black J 2008 Constructing and contesting legitimacy and accountability in polycentric regulatory regimes. Regulation & Governance 2: 137–164. 10.1111/j.1748-5991.2008.00034.x

[r5] Bock BB and van Huik MM 2007 Animal welfare: The attitudes and behaviour of European pig farmers. British Food Journal 109: 931–944. 10.1108/00070700710835732

[r6] Bradley A and MacRae R 2011 Legitimacy & Canadian farm animal welfare standards development: The case of the National Farm Animal Care Council. Journal of Agricultural and Environmental Ethics 24: 19–47. 10.1007/s10806-010-9240-z

[r2] British Columbia Society for the Prevention of Cruelty to Animals (BC SPCA) 2022 *Share your thoughts on dairy cow care.* https://spca.bc.ca/news/share-your-thoughts-on-dairy-cow-care/ (accessed December 4, 2023).

[r7] Cashore B 2002 Legitimacy and the privatization of environmental governance: How non-state market-driven (NSMD) governance systems gain rule-making authority. Governance 15: 503–529. 10.1111/1468-0491.00199

[r8] Cheyns E 2014 Making “minority voices” heard in transnational roundtables: The role of local NGOs in reintroducing justice and attachments. Agriculture and Human Values 31: 439–453. 10.1007/s10460-014-9505-7

[r9] Coffey A 2012 Analyzing documents. In: Flick U (ed) The SAGE Handbook of Qualitative Data Analysis pp 367–397. 10.4135/9781446282243.n25

[r10] Costa JHC, Cantor MC, Adderley NA and Neave HW 2019 Key animal welfare issues in commercially raised dairy calves: Social environment, nutrition, and painful procedures. Canadian Journal of Animal Science 99: 649–660. 10.1139/cjas-2019-0031

[r11] DFC n.d.a. *How it Works: Overview.* https://www.dairyfarmers.ca/proaction/how-it-works/overview (accessed October 30, 2023).

[r12] DFC n.d.b *Our Progress: Continuous improvement.* https://www.dairyfarmers.ca/proaction/our-progress (accessed October 30, 2023).

[r13] DFC 2018 *proAction Documenting Progress: 2017–2018.* https://www.dairyfarmers.ca/Media/Files/proaction/progress-report-en.pdf (accessed 28 February 2025).

[r14] DFC 2023a *Fact Sheet: Cattle Assessments.* https://www.dairyfarmers.ca/Media/Files/proaction/Cattle_Assessments-FS-Aug2023-EN.pdf (accessed 28 February 2025).

[r15] DFC 2023b *proAction Reference Manual.* https://www.dairyfarmers.ca/Media/Files/proaction/2023_dfc_proaction_workbook_en.pdf (accessed 28 February 2025).

[r16] DeVries T, Vasseur E, Duffield T, Weary DM, Winder C, Wiens D and ACER Consulting 2020 *Code of Practice for the Care and Handling of Dairy Cattle: Review of Scientific Research on Priority Issues.* https://www.nfacc.ca/pdfs/codes/scientists-committee-reports/Dairy%20Cattle%20SC%20Report%202020.pdf (accessed 28 February 2025).

[r17] Elliott L 2012 Legality and legitimacy: The environmental challenge. In: Falk R, Juergensmeyer M and Popovski V (eds) Legality and Legitimacy in Global Affairs pp 365–387. Oxford University Press: Oxford, UK.

[r18] FARM n.d.a. *FARM Animal Care Version 5 Development.* https://nationaldairyfarm.com/dairy-farm-standards/animal-care/animal-care-version-5-development/ (accessed September 17, 2024).

[r19] FARM n.d.b. *FARM Annual Report.* https://nationaldairyfarm.com/year-in-review/ (accessed November 8, 2023).

[r20] FARM n.d.c. *How to Report Animal Abuse.* https://nationaldairyfarm.com/dairy-farm-standards/animal-care/how-to-report-animal-abuse/ (accessed September 17, 2024).

[r21] FARM 2016 *2016 Year in Review.* https://nationaldairyfarm.com/resource/2016-year-in-review/ (accessed November 21, 2023).

[r22] FARM 2020 *FARM Program Recognized Again for International Quality Certification.* https://nationaldairyfarm.com/news_post/news_post-farm-program-recognized-again-for-international-quality-certification/ (accessed November 21, 2023).

[r23] FARM 2022 *2022 Year in Review.* https://nationaldairyfarm.com/wp-content/uploads/2023/03/2022-Year-in-Review_Final_SinglePage-1.pdf (accessed 28 February 2025).

[r24] FARM 2023a *Animal Care Task Force Recommendations for Version 5.0 of the FARM Animal Care Program: Public Comment Period Summary.* https://nationaldairyfarm.com/wp-content/uploads/2023/03/FARM_AC-Standards-Open-Comment-Period-Summary_Formatted.pdf (accessed 28 February 2025).

[r25] FARM 2023b *FARM V5 Town Hall [March 31, 2022]* [Video]. https://www.youtube.com/watch?v=RKX8NSH7rgg (accessed 28 February 2025).

[r26] FARM 2024a *Animal Care Reference Manual.* https://nationaldairyfarm.com/wp-content/uploads/2024/09/FARM-14787-2023-Animal-Care-Standards-Reference-Manual.pdf (accessed 28 February 2025).

[r27] FARM 2024b *Animal Care Evaluation Preparation Guide Version 5.* https://nationaldairyfarm.com/wp-content/uploads/2024/03/FARM-14125-2023-Animal-Care-V5-Evaluation-Prep-Guide-Update.pdf (accessed 28 February 2025).

[r28] Finardi C, Pellegrini G and Rowe G 2012 Food safety issues: From enlightened elitism towards deliberative democracy? An overview of EFSA’s “Public Consultation” instrument. Food Policy 37: 427–438. 10.1016/j.foodpol.2012.03.005

[r29] Fraser D and Koralesky K 2017 Assuring and verifying dairy cattle welfare. In: Beede DK (ed) Large Dairy Herd Management pp 993–1004. American Dairy Science Association: USA.

[r30] Fraser D, Koralesky KE and Urton G 2018 Toward a harmonized approach to animal welfare law in Canada. Canadian Veterinary Journal 59: 293–302. http://www.ncbi.nlm.nih.gov/pmc/articles/pmc5819020/ (accessed 28 February 2025). PMC5819020 29559748

[r31] Fraser D, Weary DM, Pajor EA and Milligan, BN 1997 A scientific conception of animal welfare that reflects ethical concerns. Animal Welfare 6: 187–205. 10.1017/S0962728600019795

[r32] Forest Stewardship Council (FSC) 2022 *Voting.* https://ga.fsc.org/en/members-resources/voting (accessed February 23, 2024).

[r33] Fuchs D, Kalfagianni A, Clapp J and Busch L 2011b Introduction to symposium on private agrifood governance: Values, shortcomings and strategies. Agriculture and Human Values 28: 335–344. 10.1007/s10460-011-9310-5

[r34] Fuchs D, Kalfagianni A and Havinga T 2011a Actors in private food governance: The legitimacy of retail standards and multistakeholder initiatives with civil society participation. Agriculture and Human Values 28: 353–367. 10.1007/s10460-009-9236-3

[r35] Fulponi L 2006 Private voluntary standards in the food system: The perspective of major food retailers in OECD countries. Food Policy 31: 1–13. 10.1016/j.foodpol.2005.06.006

[r36] Government of Canada 2023 *Milk pooling agreements.* https://cdc-ccl.ca/en/node/661 (accessed September 17, 2024).

[r37] Guest G, MacQueen K and Namey E 2012 Applied Thematic Analysis. SAGE Publications. 10.4135/9781483384436

[r38] Hahn R and Weidtmann C 2016 Transnational governance, deliberative democracy, and the legitimacy of ISO 26000: Analyzing the case of a global multistakeholder process. Business & Society 55: 90–129. 10.1177/0007650312462666

[r39] Hårstad RMB 2023 The politics of animal welfare: A scoping review of farm animal welfare governance. Review of Policy Research 41: 679–702. 10.1111/ropr.12554

[r40] Henson S 2011 Private agrifood governance: Conclusions, observations and provocations. Agriculture and Human Values 28: 443–451. 10.1007/s10460-011-9309-y

[r41] Ida JA, Wilson WM, Nydam DV, Gerlach SC, Kastelic JP, Russell ER, McCubbin KD, Adams CL and Barkema HW 2023 Contextualized understandings of dairy farmers’ perspectives on antimicrobial use and regulation in Alberta, Canada. Journal of Dairy Science 106: 547–564. 10.3168/jds.2021-21521 36424321 PMC10957287

[r42] ISO/IEC 2020 *ISO/IEC 17000:2020(en) Conformity assessment—Vocabulary and general principles.* https://www.iso.org/obp/ui/#iso:std:iso-iec:17000:ed-2:v2:en (accessed February 23, 2024).

[r43] Johnson C, Dowd TJ and Ridgeway CL 2006 Legitimacy as a social process Annual Review of Sociology 32: 53–78. 10.1146/annurev.soc.32.061604.123101

[r44] Karppinen K and Moe H 2012 What we talk about when we talk about document analysis. In: Just N and Puppis M (eds) Trends in Communication Policy Research: New Theories, Methods and Subjects pp 177–194. 10.2307/j.ctv36xvj36.12

[r45] Koppell JGS 2007 Global governance organizations: Legitimacy and authority in conflict. Journal of Public Administration Research and Theory 18: 177–203. 10.1093/jopart/mum041

[r46] Kruuse M, Tangbæk KR, Jespersen K and Gallemore C 2019 Navigating input and output legitimacy in multi-stakeholder initiatives: Institutional stewards at work. Sustainability 11: 6621. 10.3390/su11236621

[r47] Kusnezowa D and Vang J 2021 Creating legitimacy in the ISO/CEN Standard for Sustainable and Traceable Cocoa: An exploratory case study integrating normative and empirical legitimacy. Sustainability 13: 12907. 10.3390/su132212907

[r48] Lacy-Nichols J and Williams O 2021 “Part of the solution”: Food corporation strategies for regulatory capture and legitimacy. International Journal of Health Policy Management 11: 845–856. 10.34172/ijhpm.2021.111 PMC9309978 34634883

[r49] Lindgren KO and Persson T 2010 Input and output legitimacy: Synergy or trade-off? Empirical evidence from an EU survey. Journal of European Public Policy 17: 449–467. 10.1080/13501761003673591

[r50] Maciel CT and Bock B 2013 Modern politics in animal welfare: The changing character of governance of animal welfare and the role of private standards. The International Journal of Sociology of Agriculture and Food 20: 219–235.

[r51] Mena S and Palazzo G 2012 Input and output legitimacy of multi-stakeholder initiatives. Business Ethics Quarterly 22*:* 527–556. 10.5840/beq201222333

[r52] Mench JA 2008 Farm animal welfare in the U.S.A.: Farming practices, research, education, regulation, and assurance programs. Applied Animal Behaviour Science 113: 298–312. 10.1016/j.applanim.2008.01.009

[r53] Mills KE, Koralesky KE, Weary DM and von Keyserlingk MAG 2020 Dairy farmer advising in relation to the development of standard operating procedures. Journal of Dairy Science 103: 11524–11534. 10.3168/jds.2020-18487 32981724

[r54] Moog S, Spicer A and Böhm S 2015 The Politics of multi-stakeholder initiatives: The crisis of the Forest Stewardship Council. Journal of Business Ethics 128: 469–493. 10.1007/s10551-013-2033-3

[r55] Neuner FG 2020 Public opinion and the legitimacy of global private environmental governance. Global Environmental Politics 20: 60–81. 10.1162/glep_a_00539

[r56] NFACC n.d.a *About NFACC.* https://www.nfacc.ca/about-nfacc (accessed October 15, 2023).

[r57] NFACC n.d.b. *Brief History of the Codes.* https://www.nfacc.ca/brief-history-of-the-codes (accessed October 27, 2023).

[r58] NFACC n.d.c. *Dairy Code Public Comment Period Quantitative Analysis Summary.* https://www.nfacc.ca/pdfs/codes/public-comment-periods/dairy-cattle/Dairy%20Code%20Public%20Comment%20Period%20Quantitative%20Analysis%20Summary%20V_2.pdf (accessed 28 February 2025).

[r59] NFACC n.d.d. *Development process for Codes of Practice for the Care and Handling of Farm Animals.* https://www.nfacc.ca/code-development-process (accessed October 27, 2023).

[r60] NFACC n.d.e. *Project achievement reports.* https://www.nfacc.ca/achievement-reports (accessed December 3, 2023).

[r61] NFACC n.d.f. *Q&A on Public Comment Periods.* https://www.nfacc.ca/frequently-asked-questions (accessed October 26, 2023).

[r62] NFACC n.d.g. *The Animal Care Assessment Framework.* https://www.nfacc.ca/acaf-development-process (accessed October 15, 2023).

[r63] NFACC n.d.h *Your Guide to the Public Comment Period.* https://www.nfacc.ca/public-comment-period (accessed October 26, 2023).

[r64] NFACC 2019 *At-a-glance: Dairy Cattle Survey Results.* https://www.nfacc.ca/pdfs/EN_FinalDairyReport-19Sept2019_docx.pdf (accessed 28 February 2025).

[r65] NFACC 2023a *The Code of Practice for the Care and Handling of Dairy Cattle.* https://www.nfacc.ca/codes-of-practice/dairy-cattle/code2023 (accessed October 24, 2023).

[r66] NFACC 2023b *What We Heard and How We Addressed It: Updating Canada’s Code of Practice for the Care and Handling of Dairy Cattle.* https://www.nfacc.ca/pdfs/codes/what-we-heard/WWH_Farmed_Dairy_23_Final.pdf (accessed 28 February 2025).

[r67] Olmos-Vega FM, Stalmeijer RE, Varpio L and Kahlke R 2023 A practical guide to reflexivity in qualitative research: AMEE guide no. 149. Medical Teacher 45: 241–251. 10.1080/0142159X.2022.2057287 35389310

[r68] Pattberg P 2006 Private governance and the South: Lessons from global forest politics. Third World Quarterly 27: 579–593. 10.1080/01436590600720769

[r69] Rink KA, Turk P, Archibeque-Engle SL, Wilmer H, Ahola JK, Hadrich JC and Roman-Muniz IN 2019 Dairy producer perceptions of the Farmers Assuring Responsible Management (FARM) Animal Care Program. *Journal of Dairy Science* 102: 11317–11327. 10.3168/jds.2019-16859 31563309

[r70] Ritter C, Mills KE, Weary DM and von Keyserlingk MAG 2020 Perspectives of western Canadian dairy farmers on the future of farming. Journal of Dairy Science 103: 10273–10282. 10.3168/jds.2020-18430 (accessed 28 February 2025). 32952024

[r71] Roche S, Renaud D and Saraceni J 2022 *FARM Animal Care Version 5.0 Development Survey Summary Report.* https://nationaldairyfarm.com/wp-content/uploads/2022/09/FARM_Animal-Care_Version-5_Survey-Report_012622.pdf (accessed 28 February 2025).

[r72] Robbins JA, Franks B, Weary DM and von Keyserlingk MAG 2016 Awareness of ag-gag laws erodes trust in farmers and increases support for animal welfare regulations. Food Policy 61: 121–125. 10.1016/j.foodpol.2016.02.008

[r73] Rudder CE 2008 Private governance as public policy: A paradigmatic shift. The Journal of Politics 70: 899–913. 10.1017/S002238160808095X

[r74] Ryland D 2018 Animal welfare governance: GLOBALG.A.P. and the search for external legitimacy. Journal of Environmental Law 30: 453–482. 10.1093/jel/eqy015

[r75] Sankoff P 2019 Canada’s experiment with industry self-regulation in agriculture: Radical innovation or means of insulation? Canadian Journal of Comparative and Contemporary Law 5: 229–348. https://www.cjccl.ca/wp-content/uploads/2020/11/Sankoff.pdf (accessed 28 February 2025).

[r76] Schmidt VA 2013 Democracy and legitimacy in the European Union revisited: Input, output *and* ‘throughput.’ Political Studies 61: 2–22. 10.1111/j.1467-9248.2012.00962.x

[r77] Schmidt VA 2020 Conceptualizing legitimacy: Input, output, and throughput. In: Schmidt VA (ed) Europe’s Crisis of Legitimacy pp 25–55. Oxford University Press: Oxford, UK. 10.1093/oso/9780198797050.003.0002

[r78] Schmidt VA and Wood M 2019 Conceptualizing throughput legitimacy: Procedural mechanisms of accountability, transparency, inclusiveness and openness in EU governance. Public Administration 97: 727–740. 10.1111/padm.12615

[r79] Sharma LL, Teret SP and Brownell KD 2010 The food industry and self-regulation: Standards to promote success and to avoid public health failures. American Journal of Public Health 100: 240–246. 10.2105/AJPH.2009.160960 20019306 PMC2804645

[r80] Smith TM and Fischlein M 2010 Rival private governance networks: Competing to define the rules of sustainability performance. Global Environmental Change 20: 511–522. 10.1016/j.gloenvcha.2010.03.006

[r81] Spain C, Freund D, Mohan-Gibbons H, Meadow R and Beacham L 2018 Are they buying it? United States consumers’ changing attitudes toward more humanely raised meat, eggs, and dairy. Animals 8: 128. 10.3390/ani8080128 30044402 PMC6116027

[r82] Spooner J 2017 *National Farm Animal Care Council Public Comment Period Review: Final Report.* National Farm Animal Care Council. https://www.nfacc.ca/pdfs/nfacc_pcp_final_report_2018_en.pdf (accessed 28 February 2025).

[r83] Suchman MC 1995 Managing legitimacy: Strategic and institutional approaches. The Academy of Management Review 20: 571–610. https://www.jstor.org/stable/258788 (accessed 28 February 2025).

[r84] Suddaby R, Bitektine A and Haack P 2017 Legitimacy. Academy of Management Annals 11: 451–478. 10.5465/annals.2015.0101

[r85] Tepalagul N and Lin L 2015 Auditor independence and audit quality: A literature review. Journal of Accounting, Auditing, & Finance 30: 101–121. 10.1177/0148558X14544505

[r86] Thomas JC 1990 Public involvement in public management: Adapting and testing a borrowed theory. Public Administration Review 50: 435–445. 10.2307/977079

[r87] United States Department of Agriculture (USDA) 2005 Cooperatives in the dairy industry. https://www.rd.usda.gov/files/cir1-16.pdf (accessed September 17 2024).

[r88] Uzea AD, Hobbs JE and Zhang J 2011 Activists and animal welfare: Quality verifications in the Canadian pork sector. Journal of Agricultural Economics 62: 281–304. 10.1111/j.1477-9552.2011.00297.x

[r89] von Keyserlingk MAG and Weary DM 2017 A 100-Year Review: Animal welfare in the Journal of Dairy Science—The first 100 years. Journal of Dairy Science 100: 10432–10444. 10.3168/jds.2017-13298 29153174

[r90] Walker J and von Keyserlingk MAG 2018 Providing assurance that cattle have a reasonably good life. In: Engle TE, Klingborg DJ and Rollin BE (eds) The Welfare of Cattle pp 109–123. CRC Press: Boca Raton, FL, USA. 10.1201/b21911-12

[r91] Webster J 2005 The assessment and implementation of animal welfare: Theory into practice. Revue Scientifique et Technique/Office International des Epizooties 24: 723–734. https://pubmed.ncbi.nlm.nih.gov/16358522/16358522

